# Medical Bureau Pan-American Exposition

**Published:** 1903-05

**Authors:** 


### Medical Bureau Pan-American Exposition.

DIRECTOR General Buchanan of the Pan-American Exposition, presented a very comprehensive report of his labors to the directors of the Exposition Company, and portions of it were printed in the daily newspapers. That portion, however, which is of most interest to the medical profession was not printed and the JOURNAL presents it herewith. After referring generally to various matters all closely allied to the department of medicine Mr. Buchanan says:

The report of the Medical Director of the Exposition, and that of the Commandant of Police, hereto attached respectively as Exhibits "E" and "F", having been separately printed and given to the public, are sufficiently well known to make any special reference to either unnecessary, but I cannot, in justice to both of the subjects involved, omit expressing the belief that the verdict, of all who observed the operations of both organisations, was that they were superior in every feature to those of a similar character in any of the previous expositions in our country. They were certainly more economically administered than any of those to which I refer and gave equal if not better results.

The part taken by the Medical Bureau of the Exposition in the immediate care and attention given President McKinley after the fatal attempt made on his life in Music Temple has made that bureau of the exposition a part of the permanent history of our country, and every one connected with the exposition cannot but feel proud of its conduct and of the services rendered by it at that critical moment. Certainly no word of mine could add to the high reputation so justly awarded by all to the distinguished medical director of the exposition, Dr. Roswell Park, but I may be permitted to express my great appreciation of the efficient, thorough and excellent service given the exposition by him and by all those associated with him in the discharge of the duties that belonged to that bureau.

During the life of the exposition and since the dimming of its myriad of lights, the JOURNAL has had occasion to refer to the excellence of organisation of the medical department and the very thorough manner in which this very important branch of administration was conducted. It has also printed the official report, which showed that Dr. Park achieved results which were never before even approached by the medical management of any other exposition. His attention to detail and the splendid efficiency of the staff which he chose to assist him in the work are in a measure responsible for this. A good general selects able lieutenants. At no time did Dr. Park relax his vigilance and to this fact, with his splendid executive ability, is due the remarkable record of the medical department of the Pan-American Exposition,—a record which stands out in the world's history of expositions, very nearly flawless in every respect. Mr. Buchanan was not at all extravagant in his praise of the medical bureau, for a record was made at the Pan-American Exposition which expositions of the future will find it hard to equal, if not impossible to excel.

